# Unveiling the gut: microbiota and derived metabolites’ role in the rising tide of precocious puberty

**DOI:** 10.3389/fmicb.2026.1766234

**Published:** 2026-03-25

**Authors:** Anqi Zhang, Xuesong Wang, Zhanzhao Wang, Lili Zhang, Xiaohe Zhu, Yuxin Zhu, Yongmei Liu, Chenglong Zhu, Donghua Xu, Honggang Wang, Lu Zhao

**Affiliations:** 1 Department of Medical Research Center, Weifang People's Hospital, Shandong Second Medical University, Weifang, China; 2 School of Medical Laboratory, Shandong Second Medical University, Weifang, China; 3 Clinical Laboratory, Weifang People's Hospital, Shandong Second Medical University, Weifang, China; 4 Department of Obstetrics, Weifang People's Hospital, Shandong Second Medical University, Weifang, China; 5 Shandong Laibo Biotechnology Co., Ltd., Jinan, China; 6 Department of Biostatistics, School of Public Health, Cheeloo College of Medicine, Shandong University, Jinan, China

**Keywords:** endocrine, gut microbiota, gut-brain axis, metabolites, precocious puberty

## Abstract

With the rising global incidence of precocious puberty, understanding its pathogenesis is crucial. The gut microbiota, a key regulator of host physiology, has emerged as a potential contributor. While microbial alterations are observed during puberty and dysbiosis is associated with its early onset, definitive mechanisms are unclear. This review examines the role of gut microbiota and derived metabolites in precocious puberty through its effects on endocrine, immune, metabolic, and gut-brain axis. By integrating existing research, the purpose of this review is to provide a conceptual framework and highlight prospects for novel diagnostics and therapeutics.

## 1 Introduction

Precocious puberty is defined by the premature of secondary sexual characteristics in children. Diagnostic criteria typically include breast development or menarche in girls before ages 8 and 10, respectively, and pubertal onset in boys before age 9 (Latronico et al., 2016). Epidemiological studies have documented an increasing global prevalence of precocious puberty, with registry-based data indicating that overall prevalence varies widely—ranging from 37 to 935.1 per 100,000 girls and 0.46 to 37.4 per 100,000 boys, while incidence estimates span 1.123 to 489.3 per 100,000 girls and 0.096 to 22.4 per 100,000 boys (Zhang et al., 2025). The incidences of precocious puberty have increased several times in different countries: in Denmark, it increased approximately sixfold among girls between 1998 and 2017; in South Korea, the annual incidence rate rose by 83.3 times in boys and 15.9 times in girls between 2008 and 2020; and in China, recent surveys have shown that the prevalence rate among girls has reached as high as 5.01 to 7.87%. This concerning trend is attributable to multiple factors involving genetic predisposition, exposure to endocrine-disrupting chemicals, shifts in nutritional status, and psychosocial stressors (Zhang et al., 2025; Kim et al., 2019; Han et al., 2022; Bräuner et al., 2020; Kang et al., 2023; Cassio et al., 2024; Calcaterra et al., 2021; Calcaterra et al., 2023). Etiologically, precocious puberty is categorized as central precocious puberty (CPP), peripheral precocious puberty, or partial precocious puberty, with CPP accounting for approximately 80% of cases (Chen and Eugster, 2015). The present review will primarily focus on CPP.

The onset of CPP is triggered by the activation of Kisspeptin neurons, which stimulate gonadotropin-releasing hormone (GnRH) neurons to initiate pulsatile GnRH secretion. This pulsatile GnRH then stimulates the pituitary gland to secrete luteinizing hormone (LH) and follicle-stimulating hormone (FSH). In males, under the synergistic effects of LH and FSH, testicular volume increases. Specifically, LH stimulates Leydig cells to produce testosterone, while FSH supports Sertoli cell function, ultimately facilitating spermatogenesis. In females, FSH is primarily responsible for promoting follicular development, and the LH surge triggers ovulation, consequently leading to the development of secondary sexual characteristics (Calcaterra et al., 2022). However, the cause of premature hypothalamic–pituitary-gonadal axis (HPGA) activation remains unknown in approximately 90% of CPP cases (Cisternino et al., 2000). While monthly gonadotropin-releasing hormone analogs (GnRHa) injections constitute the standard treatment to suppress the release of gonadotropins and sex hormones (Luo, 2015), this approach is hampered by poor tolerability and potential side effects. Moreover, CPP can impair psychological well-being, contributing to anxiety and low self-esteem. Consequently, there is an imperative need to elucidate the underlying mechanisms of CPP and to develop less invasive diagnostic and therapeutic alternatives.

The gut microbiota (GM), a complex microbial community inhabiting the human intestinal tract (Vemuri et al., 2018), plays essential roles in digestion, immune regulation, and metabolism. Accumulating evidence indicates that GM composition is closely linked to overall health. Notably, the emergence of sex-specific GM alterations during the onset of puberty suggests a potential connection to sexual maturation (Koenig et al., 2011; Markle et al., 2013; Dong et al., 2019; Wang L. et al., 2024; Liu et al., 2024; Yue and Zhang, 2024; Bao et al., 2025; Frăsinariu et al., 2025). Mechanistically, the GM can modulate central nervous system through the gut-brain axis (Wang Q. et al., 2023), and influence the endocrine system via derived metabolites, including short-chain fatty acids (SCFAs) and amino acids, potentially regulating sex hormone production (Sudo, 2019). This review aims to summarize the association between GM and CPP, generalize its potential mechanisms, thereby underscoring the clinical imperative for early intervention. Deciphering the role of GM in sexual maturation is essential for unraveling the etiology of CPP and may yield novel therapeutic approaches (Argente et al., 2023).

### 1.1 Gut microbiota and derived metabolites in child development

#### 1.1.1 Alteration of gut microbiota during puberty

The GM undergoes a developmental succession, characterized by dynamic shifts in its diversity and structural composition throughout host growth and development, particularly during childhood (Calcaterra et al., 2022; Stewart et al., 2018; Yatsunenko et al., 2012; Hollister et al., 2015).

The early-life period (ages 0–3) represents a critical window for the assembly of the GM, characterized by a trajectory of steadily increasing taxonomic diversity from an initially low baseline (de Muinck and Trosvik, 2018). At the phylum level, the initial dominance of Proteobacteria and Firmicutes is progressively supplanted by a rising prominence of Bacteroidetes and a relative decline in Actinobacteria. This shift is also observed at the family level, as Bacteroidaceae, Prevotellaceae, and Ruminococcaceae show a marked gradual increase in relative abundance from birth to age three. Notably, Ruminococcaceae levels demonstrates a strong positive correlation with host age (Arrieta et al., 2014). Delivery mode strongly shapes initial microbial colonization (Stewart et al., 2018). Cesarean section-delivered infants exhibit elevated Proteobacteria and delayed Bacteroides colonization, whereas vaginally delivered infants acquire a microbiota richer in Bacteroides and more diverse overall. These delivery-related differences generally fade by 12 months.

In the preschool period (ages 3–4), GM diversity remains lower than in adults, and Bifidobacterium continues to be a dominant genus. Although Bifidobacterium remains abundant. This genus predominates during infancy and gradually declines with age. This developmental trajectory continues into later childhood (aged 7–12), that is higher levels of Bifidobacteria and Faecalibacterium, whereas adults exhibit increased abundances of Bacteroides (Yatsunenko et al., 2012; Hollister et al., 2015).

The GM undergoes a progressive increase in complexity throughout childhood, driven by both innate (e.g., delivery mode, genetics) and acquired (e.g., diet, environment) factors. There is a close bidirectional interaction between the onset of puberty and the GM. Sex hormones not only drive the physiological changes of puberty but also profoundly influence the composition of the gut microbial community. Conversely, the GM and its metabolites regulate the bioavailability and signaling of sex hormones through multiple pathways. As sex hormone levels rise during puberty, the GM begins to exhibit sex-specific differences, which in turn affect host hormonal homeostasis, forming a complex endocrine–microbiota regulatory network (Calcaterra et al., 2022). Although these dynamic changes are intrinsically linked to adolescent development (Yuan et al., 2020), the specific mechanisms of microbiota-hormone crosstalk during puberty await further investigation.

#### 1.1.2 Physiological functions of microbial metabolites

GM metabolites, such as SCFAs, bile acids (BAs), and amino acids, are critical signaling molecules that regulate host metabolism and maintain intestinal barrier integrity. Through these roles, they mediate a crucial crosstalk between gut microbes and host cells, which is fundamental for immune homeostasis. This interaction not only exerts a profound influence on development and overall health but also helps maintain immune balance by enhancing the epithelial barrier, regulating immune cell activity, and modulating pro- and anti-inflammatory responses. Conversely, microbial dysbiosis can disrupt this delicate balance, leading to dysregulated immune signaling and the development of various immune-related disorders (Table 1).

1) Short-chain fatty acids

**Table 1:** Physiological functions of GM derived metabolites.

Metabolite	Primary function	Disease	Role and mechanism	Reference
SCFAs	Immune regulation	IBD	SCFAs alleviate inflammatory damage in IBD by inhibiting the TLR signaling pathway and reducing NLRP3 inflammasome activation. They also inhibit the proliferation of pro-inflammatory CD4^+^ T cells, promote the differentiation of colonic mucosal Tregs, and stimulate IL-10 secretion, thereby improving intestinal immune homeostasis.	Zhang et al. (2022), Parada Venegas et al. (2019), Lee et al. (2022), Lin et al. (2023)
SCFAs	Metabolic regulation	Obesity	Acetate and propionate increase the secretion of GLP-1 and PYY by activating GPR41/43 receptors, thereby reducing appetite; they also promote fatty acid oxidation in the liver and muscles and inhibit *de novo* lipogenesis.	Jiao et al. (2020), Wei et al. (2021), Cheng Z. et al. (2022), Chambers et al. (2015)
SCFAs	Blood glucose regulation	Type II diabetes	Improves glucose homeostasis by enhancing intestinal barrier function through inducing GLP-1 and PYY production in the gut and upregulating tight junction proteins (occludin, ZO-1) and mucin (MUC2) expression.	Xia et al. (2024), Mandaliya and Seshadri (2019), Birkeland et al. (2020), Yao et al. (2020), Gu et al. (2023)
Bile acid	Metabolic regulation	Obesity	The bile acid receptor TGR5 is found in both brown adipose tissue (BAT) and muscle. When activated, it increases energy expenditure by converting dormant thyroxine (T4) to active triiodothyronine (T3).	Wei et al. (2020), Castellanos-Jankiewicz et al. (2021), Xie et al. (2021)
Bile acid	Blood glucose regulation	Type II diabetes	Activation of bile acid receptors FXR and TGR5 stimulates the release of intestinal GLP-1, which promotes insulin secretion and contributes to the management of insulin resistance and type 2 diabetes through their insulin-sensitizing effects.	Wu et al. (2021), Schlicht et al. (2025), Chen Y. et al. (2022), McMurdie et al. (2022), Sinha et al. (2020)
Bile acid	Anti-inflammatory	IBD	Activation of bile acid receptors FXR and TGR5 exerts anti-inflammatory effects by suppressing the activity of pro-inflammatory transcription factors AP-1 and NF-κB.	Sinha et al. (2020), Di Ciaula et al. (2023); Wilson et al. (2020), Zhou et al. (2023)
Tryptophan	Nervous system regulation	Depression and anxiety	Tryptophan serves as the precursor for 5-HT synthesis. A reduction in 5-HT levels leads to inadequate activation of postsynaptic 5-HT receptors, thereby diminishing the inhibition of excessive neuronal excitation.	Pan et al. (2025), Ali et al. (2021), Maxwell et al. (2019), Ishikawa and Shiga (2017)
Neurotransmitter	Immune regulation	Autoimmune diseases, IBD	Neurotransmitters can inhibit the expression of MHC class II and the antigen-presenting capacity of macrophages. They also reduce the production of pro-inflammatory cytokines, such as IL-6 and TNF-α, from macrophages and lymphocytes.	Kubera et al. (2005), Nakano et al. (2011), Maes et al. (2012)

SCFAs derived from the GM fermentation of dietary fibers exert diverse physiological effects, including immunomodulatory, anti-inflammatory, metabolism regulation, and potential protection of the cardiovascular, hepatic, and nervous systems (Murugesan et al., 2018; Frost et al., 2014; Tolhurst et al., 2012).

SCFAs modulate antigen-specific adaptive immune responses by inhibiting the migration and activation of dendritic cells and promoting T cell differentiation (Dupraz et al., 2021; Yang et al., 2020). The immunomodulatory effects of SCFA are primarily mediated via the activation of G protein-coupled receptors, which are pivotal in the regulation of immune and metabolic pathways (Liu et al., 2022; Cait et al., 2018; Kim and Kim, 2017). SCFAs downregulate pro-inflammatory factors such as interleukin-6 (IL-6) and transforming growth factor-α (TNF-α), while enhancing the secretion of anti-inflammatory factors including interleukin-10 (IL-10), transforming growth factor-β (TGF-β), and membrane protein A1 (Wen et al., 2021; Wang et al., 2017). Additionally, the anti-obesity effects of SCFAs are partly attributed to acetate and propionate, which elevate circulating levels of glucagon-like peptide-1 (GLP-1), peptide YY (PYY), and leptin, leading to reduced appetite (Jiao et al., 2020; Byrne et al., 2015). Moreover, SCFAs enhance fatty acid oxidation and concurrently suppress *de novo* lipogenesis and lipolysis, further contributing to their anti-obesity effects (Ezenabor et al., 2024; den Besten et al., 2013; Guo et al., 2022; Li et al., 2023). Sex hormones are steroid lipids primarily synthesized in the gonads and regulated by the HPGA (Corradi et al., 2016). Although SCFAs are not directly involved in sex hormones synthesis, they may indirectly influence sex hormone levels via modulation of the HPGA (Lu et al., 2017).

2) Bile acids

BAs are hepatic-derived compounds secreted into the small intestine, where they participate in a GM-liver signaling axis that regulates metabolic homeostasis. The GM modulates hepatic lipid and glucose metabolism through regulation of BAs synthesis and reabsorption, and dysregulation of this axis is implicated in various metabolic disorders, including obesity and type 2 diabetes (Renga et al., 2012; Bertaggia et al., 2017; Wei et al., 2020; Castellanos-Jankiewicz et al., 2021; Cai et al., 2024; Wu et al., 2021). Additionally, BAs exhibit anti-inflammatory properties through the receptors, farnesoid X receptor (FXR) and Takeda G protein-coupled receptor 5 (TGR5). The results of mouse studies showed that hepatic knockout of FXR and TGR5 upregulate the pro-inflammatory genes such as interleukin-1 (IL-1), IL-6, and interferon-*γ* (IFN-γ), whereas FXR and TGR5 activation is known to suppress such genes by inhibiting nuclear factor-κB (NF-κB) activity (Pols et al., 2011). Beyond above roles, BAs also act as signaling molecules that directly affect GM, forming a bidirectional regulatory relationship between host BAs metabolism and the microbial community (Mancin et al., 2023).

3) Amino acids

The GM acts as a master regulator of amino acids metabolism, controlling their breakdown, synthesis, and conversion. By producing key metabolic enzymes, specific microbes not only influence amino acid availability but also generate bioactive metabolites that interface with host immunity and neurology (Cheng et al., 2023). Among the various amino acids metabolized by the GM, tryptophan has been the most extensively studied for its role in host physiology. Tryptophan, a precursor for 5-hydroxytryptamine (5-HT), is metabolized by the GM into several distinct bioactive compounds along three main pathways: the kynurenine pathway, the tryptamine pathway, and the indole derivative pathway. Notably, these metabolites exert diverse and sometimes opposing physiological effects. For instance, kynurenine is involved in immune regulation and neuroinflammation; tryptamine modulates gastrointestinal motility and can act as a neurotransmitter; while indole derivatives (such as indole-3-propionic acid and indole-3-aldehyde) primarily serve as ligands for aryl hydrocarbon receptor (AhR), playing crucial roles in maintaining intestinal barrier integrity and regulating local and systemic immune responses (Gao et al., 2025; Hu Z. et al., 2023). Therefore, through its intricate regulation of amino acid metabolism, the GM generates a diverse array of bioactive molecules that collectively modulate immune, neurological, and intestinal barrier functions. Given the critical role of these physiological systems in the neuroendocrine regulation of reproduction, the GM, through its governance of amino acid metabolic pathways, may exert a profound influence on the development and regulation of the HPGA (Wu et al., 2023).

4) Neurotransmitters

The GM extends its influences beyond host metabolism to directly regulate neurotransmitter activity through gut-brain axis. Key neurotransmitters such as GABA, dopamine, and 5-HT are predominantly synthesized in the gut from dietary precursors by microbial symbionts and enteroendocrine cells. These signaling molecules can affect central nervous system function via the gut-brain axis. Wang et al. found that hyperactivation of enterochromaffin cells boosts 5-HT release and 5-HT3R expression, modulating visceral pain sensitivity and intestinal motility (Wang et al., 2025). Furthermore, the GM supports brain inhibitory signaling via the ammonia-Gln-GABA pathway, and its disruption impairs GABAergic function, a crucial factor in chronic stress-induced depression (Wang et al., 2023). Thus, GM dysbiosis may cause neurotransmitter dysregulation, thereby linking GM imbalance to depression and anxiety (Sun et al., 2020).

### 1.2 Gut microbiota and derived metabolites in precocious puberty

#### 1.2.1 Gut microbiota abundance in precocious puberty

Cross-sectional investigations revealed significant variations in GM composition and metabolite profiles between children with CPP and healthy controls (Huang et al., 2023; Rodríguez Mazariegos et al., 2025; Huang et al., 2022). An observational study involving girls with idiopathic CPP and healthy controls, using 16S rDNA sequencing, discovered marked alterations in the abundance of several bacterial genera including *Ruminococcus*, *Bacteroides*, *Eubacterium*, *Prevotella*, and *Clostridium* (Wang et al., 2020). These findings collectively suggest that structural and functional changes in the GM may be associated with the pathophysiology of precocious puberty. Mechanistically, GM may influence puberty timing by regulating sex hormone levels and modulating the secretion of hypothalamic GnRH via the gut-brain axis, thereby affecting the HPGA (Hu S. et al., 2023; Kim et al., 2023; Zhang et al., 2019). It should be noted, however, that most current evidence relies on 16S rRNA gene sequencing, which offers limited resolution for detecting subtle microbial changes compared to shotgun metagenomic sequencing (Ng et al., 2023).

The causal relationship and underlying mechanisms by which GM may contribute to CPP remain to be fully elucidated. Further validation through integrated multi-omics approaches-particularly metagenomics and metabolomics-coupled with evidence from animal model experiments is essential. Clarifying the role of GM in the onset and progression of CPP may not only reveal novel pathogenic pathways but also provide a scientific foundation for developing non-invasive diagnostic biomarkers, such as microbial or metabolic signatures, as well as targeted therapeutic strategies.

#### 1.2.2 Mechanisms of gut microbial metabolites in precocious puberty

1) SCFAs in precocious puberty

Studies have demonstrated that SCFAs derived from GM can influence the onset of CPP in female rats by modulating the HPGA (Rodríguez Mazariegos et al., 2025). Wang et al. (2022) found GM was disordered in obesity-induced rat models of precocious puberty, characterized by reduced production of major SCFAs. Dietary supplementation with SCFAs, including acetate, propionate, butyrate, and their mixtures, attenuated GnRH release, delayed the development of the gonadal axis, and significantly downregulated Kiss1, GPR54 and PKC protein expression as well as ERK phosphorylation. These findings suggest that SCFAs may modulate CPP in rats via the Kiss1–GPR54–PKC–ERK1/2 signaling pathway. It should be noted, however, that since this study utilized a high-fat diet-induced obesity model of precocious puberty, the generalizability of these findings to non-obesity-related precocious puberty requires further investigation. Another study demonstrated that long-term high-dose intake of the low-calorie sweetener aspartame alleviated GM dysbiosis in female rats. This restoration of microbial balance subsequently modulated fecal SCFAs levels, which, by disrupting the HPGA, significantly decreased the levels of FSH, LH and estradiol (E2), ultimately delaying the onset of puberty in female rats (Lin et al., 2024; Wang Y. et al., 2024). This experimental evidence indicates that disruption of the GM can directly impact the HPGA. Collectively, these results offer valuable insights into novel therapeutic strategies for precocious puberty, positioning GM-derived SCFAs as a promising target for intervention.

2) Bile acid metabolism in precocious puberty

Primary BAs are synthesized directly from cholesterol in the liver. Upon secretion into the intestine via bile, they are metabolized by the GM and converted into secondary BAs (de Aguiar Vallim et al., 2013). Existing studies have demonstrated that BAs exert a positive effect on estrogen levels through activation of hepatic farnesoid X receptor (FXR), thereby mediating the inhibition of estrogen sulfotransferase SULT1E1. Furthermore, a bile acid combination comprising 6-ketoLCA, CDCA, and TαMCA was identified as a promising diagnostic biomarker, exhibiting favorable diagnostic performance for CPP (Wang S. et al., 2025). Moreover, studies have revealed that a substantially lower ratio of conjugated to unconjugated BAs in pre-adolescent individuals compared to adolescents during puberty, with secondary BAs levels increasing both before and after pubertal onset. Notably, early overexpression of bile acid receptor TGR5 in the arcuate nucleus of female rats has been shown to accelerate pubertal onset, as well as TGR5 mRNA expression was higher on postnatal day 21 than day 14. This effect may be mediated through crosstalk between TGR5 and kisspeptin signaling, whereby secondary BAs activate TGR5, thereby stimulating GnRH release within the hypothalamus (Vanden Brink et al., 2024). Furthermore, glycocholic acid has been reported to promote IL-22 secretion by innate lymphoid cells in the intestine, and IL-22 has been demonstrated to ameliorate polycystic ovary syndrome (PCOS) (Qi et al., 2019). Notably, both CPP and PCOS share etiological links to dysregulation of HPGA (Chiavaroli et al., 2010). Nevertheless, the direct relationship between BAs and CPP remains insufficiently explored.

3) Amino acid metabolism in precocious puberty

Amino acids serve not only as fundamental constituents of proteins, but also as critical regulators in a variety of biological processes, such as hormone synthesis and neurotransmitter creation, both of which are linked to sexual development.

Chen G. Y. et al. (2022) identified elevated levels of amino acids, including isoleucine, N-fructosyl isoleucine, indoline, phenylalanine, and tyrosine, in girls with CPP. Among these, tyrosine and phenylalanine are precursors of catecholamines, with norepinephrine playing a crucial role in regulation of GnRH neurons (Bronzi et al., 2015). Additionally, glutamate (Glu) can also regulate GnRH release and is essential for activating the HPGA (Dhandapani and Brann, 2000; Maffucci and Gore, 2009). The marked reduction in Glu levels observed in CPP girls indicates dynamic metabolic changes during disease progression, which may be due to the high consumption of Glu caused by rapid development (Wu et al., 2023). In peripheral tissues, tryptophan is converted into 5-hydroxytryptophan by tryptophan hydroxylase, and further metabolized to the neurotransmitter 5-HT (Layunta et al., 2021). As a crucial precursor molecule, tryptophan primarily exerts its effects through this metabolite, modulating the levels of GnRH in the hypothalamus, thereby influencing the timing of puberty onset. These findings directly implicate amino acids in neuroendocrine regulation of the gonadal axis, a process potentially modulated by GM to influence precocious puberty.

4) Neurotransmitter metabolism in precocious puberty

Evidence suggests that neurotransmitter metabolites such as 5-HT and dopamine can promote sexual maturation through activation of the HPGA (Sittipo et al., 2022), whereas GABA exerts an inhibitory effect on pubertal onset. Research has found that the sharp decrease in GABA levels on the afternoon before ovulation may act via GABA_A_ receptors to inhibit the subsequent LH surge (Li et al., 2015; Liu et al., 2020). Specifically, 5-HT may stimulate GnRH secretion via activation of the phospholipase C signaling pathway in the hypothalamus, thereby facilitating hormonal release. In patients with CPP, urinary levels of 5-hydroxytryptophan, the precursor of 5-HT, were significantly decreased, while metabolites of 5-HT such as 5-hydroxyindoleacetic acid and 5-hydroxyindoleamine were markedly elevated (Qi et al., 2012). These alterations suggest 5-HT metabolic pathways is closely involved in CPP, but the mechanism by which GM derived neurotransmitters affect CPP remains unclear and requires further investigation.

**Figure 1:**
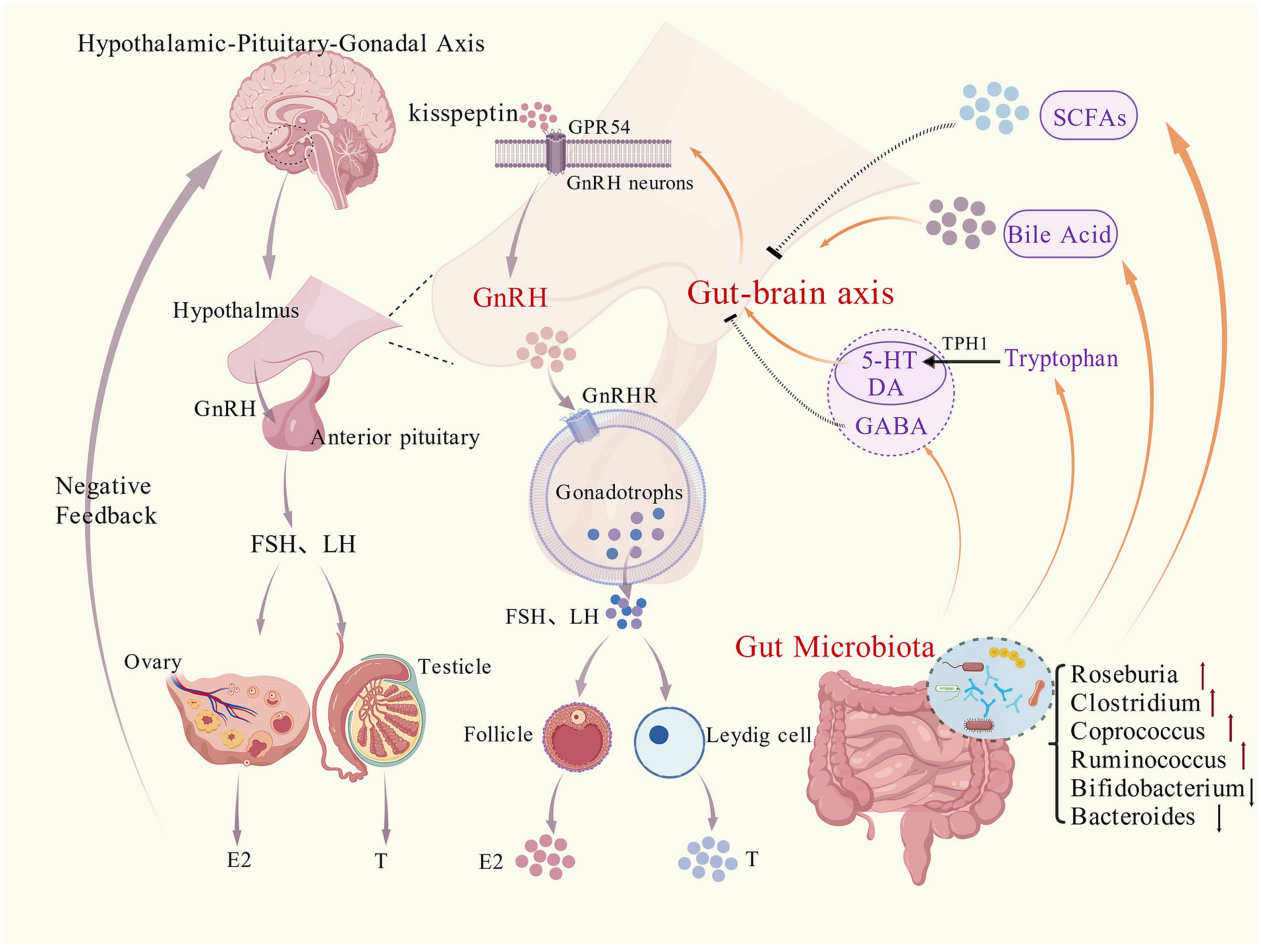
Molecular mechanisms by which gut microbiota and their derived metabolites regulate the hypothalamic–pituitary-gonadal axis (HPGA) to modulate CPP. GnRH, gonadotropin-releasing hormone; FSH, follicle-stimulating hormone; LH, luteinizing hormone; SCFAs, short-chain fatty acids; GPR, G protein-coupled receptor; PKC, protein kinase C; ERK1/2, extracellular signal-regulated kinase 1/2; TPH1, tryptophan hydroxylase 1; GABA, *γ*-aminobutyric acid; 5-HT, 5-hydroxytryptamine; DA, dopamine; E_2_, estradiol; T, testosterone. Color-coded arrows are defined as follows: Purple, HPGA regulatory processes; orange, gut-brain axis pathway processes; dashed arrows, inhibitory effects; red, increased microbiota abundance; black, decreased microbiota abundance. Figure was created by BioGDP (www.biogdp.com).

As a summary, we display a diagram of the molecular mechanism showing how GM and their metabolites can (bidirectionally) trigger CPP in Figure 1. Distinct from the previously published review by Yue and Zhang (2024), which provided a broad overview of the relationship between the GM and CPP, the characteristic of the present manuscript focuses on the mechanistic aspects of microbial metabolites. We reviewed the comprehensive contents about the interactions between specific GM-derived metabolites and the neuroendocrine system. Furthermore, we have summarized the newest findings and illustrated the updated mechanistic framework.

### 1.3 Gut microbiota intervention for precocious puberty

#### 1.3.1 Probiotic intervention

Further in-depth research elucidating the precise mechanisms is essential to developing novel, GM-targeted therapies, such as probiotic interventions utilizing specific bacteria for the prevention and management of CPP in children.

Probiotics are beneficial microorganisms that maintain host health by colonizing the human intestinal tract and improving microbial balance (Wilkins and Sequoia, 2017; Suez et al., 2019). These beneficial microbes are primarily derived from fermented foods such as yogurt and kimchi, or through dietary supplements. Commonly used probiotic strains include *Streptococcus thermophilus*, *Bifidobacterium lactis*, *Lactobacillus bulgaricus*, and *Lactobacillus acidophilus*, which are frequently employed in fermented dairy products (Tsai et al., 2022). Probiotics help maintain gut homeostasis by modulating the GM, inhibiting the proliferation of pathogenic bacteria, and enhancing intestinal barrier function (Bonder et al., 2016). Additionally, they support gut immune function, promote gut motility, and facilitate normal digestive processes (La Fata et al., 2018).

Numerous studies have revealed that probiotic beverages may delay puberty onset. In obese precocious rat models, probiotic intervention significantly reduced serum levels of triglycerides (Tg), total cholesterol (TC), LH, FSH, and E_2_, and downregulated Kiss-1 and GnRH expression. Histopathological analysis indicated probiotics ameliorated obesity-induced ovarian and uterine alterations, reducing follicular maturation and improving endometrial structure (Tsai et al., 2022; Gaskins et al., 2017; Qian et al., 2024; Yuan et al., 2023). These findings demonstrate that while obesity accelerates precocious puberty, probiotic supplementation attenuates its development by normalizing reproductive hormones and preserving gonadal morphology, suggesting potential preventive and therapeutic value.

#### 1.3.2 Prebiotic intervention

Prebiotics are non-digestible food ingredients by the host but can be fermented by the GM to promote growth and proliferation of beneficial microbes, thereby conferring health benefits to the host. Common prebiotics including fructooligosaccharides, inulin, galactooligosaccharides, and lactulose have been proven to improve diabetes, IBD, osteoarthritis of obesity and other diseases (Zhang et al., 2023; Akram et al., 2019; Schott et al., 2018). Prebiotics can be broken down into various bioactive metabolites, which promote the proliferation and metabolic activity of beneficial bacteria including *Bifidobacterium* and *Lactobacillus*. This modulation of gut microbiota composition and function has been shown to ameliorate symptoms associated with precocious puberty (Yuan et al., 2023; Floch, 2018; Guarino et al., 2020; Thiruvengadam et al., 2023).

#### 1.3.3 Fecal microbiota transplantation

Fecal Microbiota Transplantation (FMT) has been successfully used in clinical treatment of obesity, diabetes, Crohn’s disease, ulcerative colitis, autism spectrum disorder and other diseases (Unrug-Bielawska et al., 2025; Yu et al., 2022; Vatanen et al., 2024; Li et al., 2020; Vendrik et al., 2020). The results of animal experiments showed that FMT from glycyrrhizin-treated female mice significantly delayed vaginal opening time and reduced GnRH expression in recipient mice (Bo et al., 2022), which provide new experimental evidence for microbiota-targeted interventions in reproductive and pubertal development. Additionally, FMT from healthy female rats has been shown to improve polycystic ovary syndrome (Guo et al., 2016). This indicates that the GM exerts a decisive influence on the host’s gonadal function.

## 2 Conclusions and future perspectives

In summary, this review systematically consolidates current evidence linking GM dynamics to the pathogenesis of precocious puberty. It clarifies their critical roles in the endocrine, immune, and metabolic pathways that govern pubertal maturation. The study on GM is advancing toward mechanistic explanation and translational validation. Nevertheless, the causal relationship between the GM and CPP remains elusive. Which specific microbial taxa or metabolic modules are sufficient to trigger or delay HPGA activation? Through which targets do GM exert their effects on host? Thus, answering these fundamental mechanism questions will improve the development of targeted interventions on CPP. Future work should prioritize mechanistic interrogation of specific microbial taxa in GM.

From the perspective of GM regulation, the clinical application in CPP mainly involves two aspects. On one hand is in-depth investigation of therapeutic strategies. To date, probiotic interventions for CPP have primarily utilized common strains (e.g., certain Lactobacillus and Bifidobacterium species) or broad-spectrum microbial consortia, but no well-defined probiotics can currently be recommended for clinical use in CPP. There is still vast research space for designing a specific probiotic combination or engineered probiotic for clinical therapy of CPP. Mechanism-guided screening strategies also remain absent. Given that metabolite profiles vary markedly across strains, their effects on the host HPGA may differ substantially. Both in randomized controlled trial and animal models are needed to validate probiotic modulate pubertal timing. Such efforts will eventually establish a complete evidence chain–from strain to metabolite, and then to target tissue or genes, and finally to the phenotype of host. This will enable the identification of probiotic strains with clearly defined functions. On this basis, individualized intervention strategies can be developed by integrating an individual’s GM composition, genetic background, and dietary habits. On the other hand, GM biomarkers for CPP cohorts in different regions are great diagnostic value. Currently, the clinical diagnosis of CPP relies heavily on the GnRH stimulation test, bone age assessment, and hormone measurements. These methods are either invasive or involve radiation exposure. Compared with traditional invasive tests, fecal sampling is convenient, painless, and highly reproducible–attributes particularly suited to pediatric populations. Future investigations should aim to identify core microbial taxa or characteristic metabolite signatures closely linked to pubertal timing. Constructing diagnostic prediction models based on these signatures would facilitate early screening, disease monitoring, and therapeutic evaluation in high-risk populations. Such noninvasive strategies, grounded in the gut–brain axis, not only promise to alleviate physical and psychological distress in children but also equip clinicians with a practical tool for dynamic monitoring. In the future, such “gut-HPGA” diagnostic and therapeutic approaches be effectively translated from bench to bedside, offering safe and effective support for children’s developmental health.
